# Six1 Regulates MyoD Expression in Adult Muscle Progenitor Cells

**DOI:** 10.1371/journal.pone.0067762

**Published:** 2013-06-28

**Authors:** Yubing Liu, Imane Chakroun, Dabo Yang, Ellias Horner, Jieyi Liang, Arif Aziz, Alphonse Chu, Yves De Repentigny, F. Jeffrey Dilworth, Rashmi Kothary, Alexandre Blais

**Affiliations:** 1 Ottawa Institute of Systems Biology, University of Ottawa, Ottawa, Ontario, Canada; 2 Department of Biochemistry, Microbiology and Immunology, University of Ottawa, Ottawa, Ontario, Canada; 3 Ottawa Hospital Research Institute, Ottawa, Ontario, Canada; 4 Departments of Medicine, and Cellular and Molecular Medicine, University of Ottawa, Ottawa, Ontario, Canada; The Chinese University of Hong Kong, China

## Abstract

Quiescent satellite cells are myogenic progenitors that enable regeneration of skeletal muscle. One of the early events of satellite cell activation following myotrauma is the induction of the myogenic regulatory factor MyoD, which eventually induces terminal differentiation and muscle function gene expression. The purpose of this study was to elucidate the mechanism by which MyoD is induced during activation of satellite cells in mouse muscle undergoing regeneration. We show that Six1, a transcription factor essential for embryonic myogenesis, also regulates MyoD expression in muscle progenitor cells. Six1 knock-down by RNA interference leads to decreased expression of MyoD in myoblasts. Chromatin immunoprecipitation assays reveal that Six1 binds the Core Enhancer Region of *MyoD*. Further, transcriptional reporter assays demonstrate that Core Enhancer Region reporter gene activity in myoblasts and in regenerating muscle depends on the expression of Six1 and on Six1 binding sites. Finally, we provide evidence indicating that Six1 is required for the proper chromatin structure at the Core Enhancer Region, as well as for MyoD binding at its own enhancer. Together, our results reveal that MyoD expression in satellite cells depends on Six1, supporting the idea that Six1 plays an important role in adult myogenesis, in addition to its role in embryonic muscle formation.

## Introduction

Skeletal muscle is a plastic organ that can regenerate itself after injury. This property relies on the presence of resident adult stem cells termed satellite cells (reviewed in [1]). In resting adult muscle, satellite cells are quiescent and are found in low numbers positioned between the basal lamina and the myofiber sarcolemma. Muscle injury leads to the activation of satellite cells: they are released from their anatomic position, initiate several rounds of cell division, and eventually undergo myogenic differentiation to create new muscle mass [2]. Recent work has demonstrated the importance of satellite cells in muscle regeneration [3]–[6].

At the gene expression level, quiescent satellite cells are characterized by the expression of the Paired-box transcription factor Pax7 [7], and by their lack of expression of muscle structural genes. Although the messenger RNA for the myogenic regulatory factor (MRF) Myf5 transcription factor is expressed by most quiescent satellite cells, the Myf5 protein itself is absent from these cells [8], [9]. Likewise, the other proteins of this family, MyoD, myogenin and MRF4 are unexpressed in quiescent cells. Instead, MyoD expression is strongly induced early after injury, as satellite cells become activated [10], [11]. After proliferation, satellite cells initiate their terminal differentiation: MyoD activates the expression of the MRF myogenin, as well as a host of muscle function genes, and the cells undergo fusion and exit the cell cycle.

Very little is known about the mode of up-regulation of MyoD expression in satellite cells; what we know of this gene’s regulation concerns embryonic development. Three important *cis*-regulatory elements active during embryogenesis have been identified: the core enhancer region (CER), the distal regulatory region (DRR), and the proximal regulatory region (PRR, reviewed in [12]). Transgenic analyses have revealed that the CER, which is located 23 kb upstream of the transcription start site of the MyoD gene, drives expression in muscle precursor cells of the limb buds and the myotome [13], [14]. The essential role of the CER in controlling the proper temporal expression of MyoD has been demonstrated by deletion analyses: MyoD transcription is delayed by one to two days in the limb buds and branchial arches of mouse embryos lacking the enhancer, although myotomal expression, which depends on Myf5 and Pax3 expression, is not affected [15]. The DRR on the other hand, appears to drive expression of a LacZ reporter gene later, in differentiated cells of the muscle lineage, and possibly also in adult muscles [16], [17]. Deletion analyses have confirmed the important role of the DRR in adult muscle expression of MyoD, but have also revealed that the DRR is in fact necessary, albeit not sufficient, to drive full MyoD expression levels in the limb buds and branchial arches at E10.5 [18]. Finally, the PRR, which in mice has been defined as a 275-bp sequence immediately preceding the transcription start site, does not have muscle-specific activity in mammalian cells, but cooperates specifically with the DRR to confer muscle specificity [19].

Various factors that act upon these three regulatory elements have been identified. AP-1, the dimer formed by Jun and Fos family members, activates transcription of a reporter gene containing the MyoD PRR [20]. The PRR is also bound by a CCAAT-binding activity in a muscle-specific manner, and its transcriptional activity depends on a consensus Sp1 binding site. The MRFs Myf5 and MyoD themselves are suspected to act upon the CER and DRR in various developing structures, as transgenic reporter activities are diminished in MyoD^−/−^;Myf5^−/−^ embryos [21]. Although direct binding of these MRFs to the CER or DRR has not been assessed in the embryos, both elements contain E-box motifs [14], [19], and MyoD has been shown by ChIP-seq to bind to both elements in C2C12 myoblasts [22], and to bind the DRR in differentiating myoblasts [23]. The homeobox factor Msx1 represses MyoD expression and delays the execution of the myogenic program in the distal portion of the developing limb bud, by binding to the CER and inducing the formation of a repressive chromatin environment [24]–[26]. In an analogous manner, the bHLH-PAS factor Sim2 can also repress MyoD expression by binding to the CER in the developing limb bud [27]. In contrast, the homeodomain transcription factor Pitx2 is involved in the initiation of MyoD expression in the limb bud, and directly binds to the CER [28]. In the adult, binding of Bmal1 and Clock circadian bHLH-PAS transcription factors to the CER is responsible for the circadian rhythmicity of MyoD expression in skeletal muscle [29], [30]. In adult muscle regeneration, Mef2 factors and their co-regulator MASTR have been shown to bind to the DRR to activate MyoD expression in satellite cells [31]. Likewise, Serum Response Factor (SRF) can bind to the DRR, and DRR-dependent transgene expression in regenerating adult muscle depends in part on the presence of the SRF binding site [32], [33].

The Six family of homeodomain transcription factor has also been shown to play a role in regulating MyoD expression. The Six family consists of 6 members, from Six1 to Six6. They are involved in controlling the differentiation of various tissue types [34]. Six1 and Six4 are important for embryonic myogenesis: Six1^−/−^ mouse embryos have important myogenesis defects that are exacerbated in Six1^−/−^;Six4^−/−^ animals [35]–[37]. Importantly, MyoD expression is severely impaired in the absence of Six1, in the limb bud as well as in the epaxial extension of the dermomyotome and the ventral myotomal extension. In animals lacking both Six1 and Six4, a Six family factor that may partially compensate for the absence of Six1, MyoD expression is further affected and persists only in the hypaxial-ventral portion of the interlimb somites myotome. Six1^−/−^ and Six1^−/−^;Six4^−/−^ fetuses die at birth from respiratory failure and it is only recently that the role they play in adult satellite cells during regeneration has been revealed. Conditional ablation of Six1 in Pax7-expressing satellite cells leads to a regeneration failure following injury, due to myoblast differentiation impairment. MyoD expression is diminished in these cells, and this is thought to be mediated at least in part by a binding site for Six1 in MyoD’s DRR [23].

We have previously reported a genome-wide profiling of Six1 binding sites in C2C12 mouse myoblasts and myotubes. We found that Six1 and Six4 are necessary for the myogenic differentiation of these cells, and that they accomplish their function in part by activating shared target gene expression in cooperation with the MRFs [38]. We have now investigated the function of Six1 in primary adult mouse myoblasts, and focused on the role it plays in regulating the expression of MyoD.

## Materials and Methods

### Ethics Statement

Surgical procedures were performed using aseptic techniques and in complete agreement with the University of Ottawa Animal Care and Use Committee in compliance with the Guidelines of the Canadian Council on Animal Care and the Animals for Research Act. The University of Ottawa Animal Care and Use Committee approved this study.

### Muscle Injury and Immunostaining

Seven-week-old C57BL/6 female mice (Charles River, Canada) were first anesthetized with isoflurane. Both legs were shaved, and 30 µl of cardiotoxin (CTX, Latoxan, France, 10 mM in phosphate-buffered saline (PBS)) were injected into the left TA, and 30 µl of PBS were injected into the right TA muscle as control. A total of 5 mice were used for each time point to account for biological variability. Mice were sacrificed by cervical dislocation 2, 3, 4 or 7 days after cardiotoxin injection. Their TA muscles were dissected and fixed in 2% (w/v) paraformaldehyde in PBS overnight at 4°C. Muscles were then washed 2 times for 5 minutes in cold PBS (4°C), and quenched with 0.25 M glycine in PBS 2 times for 10 minutes on ice. Muscles were then incubated in 5% sucrose (in PBS) for a minimum of 6 hours, and then incubated in 20% sucrose (in PBS) overnight at 4°C. Muscles were frozen with Tissue-Tek O.C.T. (Optimal Cutting Temperature) Compound in 2-methylbutane chilled in liquid nitrogen, and stored in −80°C freezer. Muscles were cryosectioned at −28°C into 10 µm sections on Fisherbrand Superfrost Plus slides and stored at −80°C until antigen retrieval was performed.

Slides were dried at 37°C for 10 minutes, then incubated in citrate buffer (10 mM citric acid, 0.05% Tween 20 (v/v), pH 6.0) for 20 minutes at 98°C, and slides were cooled down at room temperature in citrate buffer for 20 minutes. Sections were subsequently rinsed with PBS-T (PBS, 0.1% Tween 20 (v/v)) and then permeabilized with 0.5% Triton X-100 (v/v) in PBS for 10 minutes at room temperature. Sections were rinsed again with PBS-T and blocked with blocking buffer (3% bovine serum albumin (w/v) in PBS-T) for 1 hour at room temperature (or overnight at 4°C). Sections were further blocked for 1 hour at room temperature with 3.6% (v/v) M.O.M. Mouse Ig Blocking Reagent solution in PBS (Vector Laboratories), then washed twice with PBS. Sections were incubated with primary antibodies against Six1 (1∶100 v/v rabbit anti-mouse, made in-house), MyoD (1∶100 v/v Santa Cruz, sc-32758X, 5.8A mouse monoclonal), Pax7 (1∶100 v/v Developmental Studies Hybridoma Bank) in blocking buffer for 1 hour at room temperature, and rinsed with PBS-T. Adjacent sections without primary antibodies were also prepared, to ensure the specificity of the resulting fluorescent signals. Sections were incubated with 1∶200 biotin-conjugated donkey anti-mouse IgG F(ab′) fragment (Jackson Immunoresearch) in blocking buffer for 1 hour at room temperature, and washed in PBS-T. Sections were incubated with secondary antibodies conjugated to Alexa594 (1∶1000, Invitrogen, donkey anti-rabbit IgG) and with Steptavidin-Alexa488 (1∶1000, Invitrogen) in blocking buffer for 1 hour at room temperature in the dark, and rinsed with PBS-T. Sections were rinsed with PBS, then de-ionized water, and with 70% ethanol before incubation with 0.3% Sudan Black (w/v, Sigma) in 70% ethanol for 10 minutes. Slides were washed with PBS and washed with de-ionized water. Slides were air-dried and mounted with ProLong Gold antifade reagent with DAPI (Invitrogen).

Images were acquired with a Carl Zeiss Axiovision Observer D1 microscope operated with the Axiovision Rel 4.8 software, using a 20× objective. Six images, each representing fields of 670 µm by 896 µm (0.6 mm^2^) were acquired per sample, at different locations on the muscle section. For the purpose of preparing figures, Adobe Photoshop was used to adjust levels (black and white points, but not gamma), taking care to avoid clipping pixels and applying changes to the entire image. Cell counting was performed with the ImageJ 1.45 software and the Cell Counter plug-in (W. Rasband, National Institutes of Health). Nuclei positive for either of the proteins of interest were counted in each image, and the average was calculated. The average and standard error of those counts over the 5 replicates were finally calculated.

### Cell Culture

The C2C12 cells (American Type Culture Collection) were grown in Growth Medium (GM) containing 88% Dulbecco’s Modified Eagle’s Medium (DMEM); 10% Fetal Bovine Serum (FBS); 1% L-Glutamine and 1% Penicillin/Streptomycin (P/S)) until confluent. Once confluent, the cells were induced to differentiate by replacement with Differentiation Medium (DM) containing 96% DMEM; 2% Horse Serum; 1% L-Glutamine and 1% P/S. The cells were grown in a humidified water-jacketed incubator at 37°C with 5% CO2. The C2iFRT cell line that constitutively expresses the Tet-repressor and contains a single genomic FRT recombination site insertion has been previously described [39]. A cDNA encoding N-terminally Flag-tagged mouse MyoD was cloned into the MCS of the pCDNA5/FRT/TO plasmid. The pCDNA5/FRT/TO-FL-MyoD plasmid was co-transfected with the Flp recombinase into C2iFRT cells and selected for hygromycin expression as outlined in the Flp-In system protocol (Invitrogen). Batch cultures of C2iFRT-FL-MyoD cells were then screened for their ability to differentiate to form myotubes and for MyoD expression after induction with doxycycline at a concentration of 0.5 µM. Primary myoblasts were prepared as described by Rando et al. [40] with the modifications outlined below. The primary myoblasts were isolated from 60 day old C57BL/6 female mice. *Gastrocnemius*, *tibialis anterior* (TA) and *quadriceps* muscles were pooled and digested with 0.2% Collagenase I (Sigma) and 625 µg/mL Dispase II (Roche) for 1.5–2.0 hours at 37°C. Cells were then diluted with DMEM and passed through a 70 µm nylon mesh filter (BD Falcon) to remove undigested connective tissues. Cells were rinsed twice with DMEM and resuspended in plating medium containing 90% DMEM; 10% donor equine serum and 5 ng/mL of bFGF (Peprotech). Cells were then pre-plated twice for 1 h in a 10 cm tissue culture treated dish (Corning), transferred to Matrigel (BD Biosciences) coated cell culture dishes and allowed to adhere to the plate for 48 h. Cells were subsequently maintained in DMEM (American Type Culture Collection) with 20% Fetal Bovine Serum (HyClone), 10% Donor Serum (HyClone) and 1% P/S (HyClone) supplemented with 10 ng/mL of bFGF and 2 ng/mL of bHGF (Peprotech).

### RNAi

Primary and C2C12 myoblasts were transfected with siRNA duplexes (non-silencing or Six1-specific) using Lipofectamine RNAiMax (Invitrogen), essentially as described previously [38].

### Six1 Antibody Preparation

New Zealand white rabbits were immunized with full-length histidine-tagged recombinant mouse Six1 protein, as described previously [38]. The serum was purified by running rabbit serum through a column of immobilized GST-Six1 protein (amino acids 198–248, sharing no homology to other family members), washed several times with tris-buffered saline and eluted with a solution of 0.1 M glycine pH 2.5 to elute specific anti-Six1 antibodies. Specificity was confirmed by western blot on all six full-length proteins of the murine Six family, produced in rabbit reticulocyte lysates: only Six1 is detected by the purified antibodies (data not shown).

### Western Blots

Total protein lysates were prepared by rinsing the cells in PBS and lysing in 20 mM Tris pH 6.8, 6 M urea and 0.1% SDS. Lysates were sonicated briefly and spun down to remove debris. Primary antibodies used are anti-Six1 (rabbit, home-made), anti beta-actin (mouse monoclonal, Sigma), anti-MyoD (mouse monoclonal 5.8A, Santa-Cruz) or anti-beta-tubulin (mouse monoclonal, E7, Developmental Studies Hybridoma Bank).

### ChIP Assays and ChIP-sequencing

Chromatin immunoprecipitation assays were performed as described before [38], for both C2C12 and primary myoblasts. The antibodies used were rabbit anti-Six1 [38] and normal rabbit IgG (Jackson Immunoresearch). For ChIP assays on transfected cells, a 9∶1 mixture of either wild-type or mutant reporter plasmid (described below) and puromycin resistance plasmid was transfected in C2C12 myoblasts using the polyethylenimine method [41]. Cells were selected with puromycin for 7 days, resistant clones were pooled to generate polyclones and expanded, and chromatin was prepared as described before [38]. PCR primer sequences used in ChIP are: CER-F: TGCTTCTTTCGGCCAAGTAT; CER-R: CCAACTGGCTGTGTTGTGAG; HoxD10-F: GAGAAATCGGACTCACCTTCC; HoxD10-R: CACATACCCAGGCAGAACG. For PCR to distinguish the endogenous CER and the CER transgene, primers were a- GTTGGGGGAAGGGGACAG; b- GACTCCAGGAAGGAAGAAGAGG; c- ACCCGTGACTCACAACACAG; d- TCTCCAGTGTCTACTCGAG. Quantitative PCR was performed on input chromatin from the wild-type and mutant polyclones and on a titration curve made with the pure reporter plasmid to ensure that the transgene copy numbers are comparable for both polyclones; the wild-type and mutant polyclones contain respectively 2.1 and 2.2 copies of transgene per cell (data not shown). Transgene ChIP data were analyzed as follows. First, qPCR titration curves made of input chromatin from the wild-type or the mutant polyclones were run in parallel to the ChIP samples, so “percent-of-input” values could be ascribed to each ChIP sample. Second, the percent of input values obtained with the non-specific antibody control (normal IgG) were subtracted from the percent-of-input values obtained with the other antibodies. Third, those IgG-subtracted percent-of-input values were reported as fractions of the values obtained with the wild-type CER polyclone.

ChIP-seq experiments were performed on 50 million primary myoblasts in growth phase. Chromatin was fragmented to an average size of 200 bp and immunoprecipitated using rabbit anti-Six1 or a control rabbit IgG (Jackson). An input chromatin sample (prior to immunoprecipitation enrichment) was also prepared. After purification, sequencing libraries were prepared by the McGill University and Génome Québec Innovation Centre, and sequenced at 1 sample per lane on HiSeq2000. Sequencing reads were aligned to the mm9 mouse genome assembly using Bowtie in –n mode [42], allowing 0 mismatches in the first 36 nucleotides of each read, and removing reads that align at more than one location in the genome. Picard was used to filter out replicated reads (http://picard.sourceforge.net/). SeqMonk (http://www.bioinformatics.babraham.ac.uk/projects/seqmonk/) was used to extend reads to a length of 200 bp, to normalize read counts to the total number of retained reads in each sample, and to calculate normalized read densities for each sample in contiguous, non-overlapping bins of 25 base pairs. The read density in the input sample was finally subtracted from the immunoprecipitation samples. These read densities are given as wiggle format files as Table S1. A full description of the ChIP-seq results will be published elsewhere (Y.L. and A.B., in preparation).

### EMSA

Full-length CER DNA probes were prepared by PCR amplification from wild-type or mutant plasmids, restriction digestion and fill-in with Klenow enzyme (Promega) in the presence of Cy5-labelled dCTP. Recombinant Six1 was produced in rabbit reticulocyte lysates (Promega). Gels were scanned on a Typhoon imager (GE Healthcare).

### Reporter Constructs and Reporter Assays

PCR on C2C12 genomic DNA was used to amplify the murine CER and PRR regions separately, adding restriction sites for cloning: CER-F: GACGACGCTAGCTGAGCCCCACAGCATTTGGG, CER-R: GACGACCTCGAGCCCCAGCCCTAGGCCTGAGC; PRR-F: GACGACCTCGAGTAGACACTGGAGAGGCTTGGG; PRR-R: GACGACAGATCTAGGCGCCCTGGGCTATTTATCC. The PRR was cloned upstream of the luciferase gene in pGL3-Basic (Promega) and the CER was subsequently cloned upstream of the PRR. Mutations were introduced in the CER region by sequential overlapping mutagenic PCR reactions. The wild-type CER+PRR were additionally cloned upstream of the LacZ reporter in the p1230 plasmid [43], from which the beta-globin minimal promoter had been removed by restriction and re-ligation. The CMV-Renilla luciferase plasmid (Promega) was used as an internal control. The Myogenin promoter wild-type and MEF3 site mutant constructs were described previously [38].

Mice were injected with cardiotoxin, and three days later were injected with 25 uL of a saline solution containing 12.5 ug of reporter DNA (10 ug luciferase, and 2.5 ug CMV-Renilla). The DNA was then electroporated. Four days later, the animals were sacrificed by cervical dislocation, their TA muscles were removed and quickly frozen in liquid nitrogen. The tissue was crushed to a fine powder and resuspended in passive lysis buffer (Promega). C2C12 cells were transfected using the polyethylenimine method, and harvested 48 hours after transfection, by rinsing in PBS and lysing in passive lysis buffer. Lysates were assayed for firefly and renilla luciferase activities using the Dual Luciferase assay kit (Promega). To normalize across samples, firefly luciferase activity values were divided by those of renilla luciferase. Where indicated, this ratio was further normalized by dividing by the normalized luciferase readings obtained in control conditions.

### Transgenic Reporter Mice

We tested in mouse embryos the fidelity of our mouse CER-PRR construct as a fusion with the LacZ reporter gene, by pronuclear injection of DNA using standard methods [44]. Founder embryos were harvested at 11.5 days of gestation, and were fixed and stained with X-Gal to reveal beta-galactosidase activity. A total of 15 founder embryos, from 2 separate litters, gave comparable results.

## Results

### Six1 is Expressed in Satellite Cells of Regenerating Muscle

**Figure 1:**
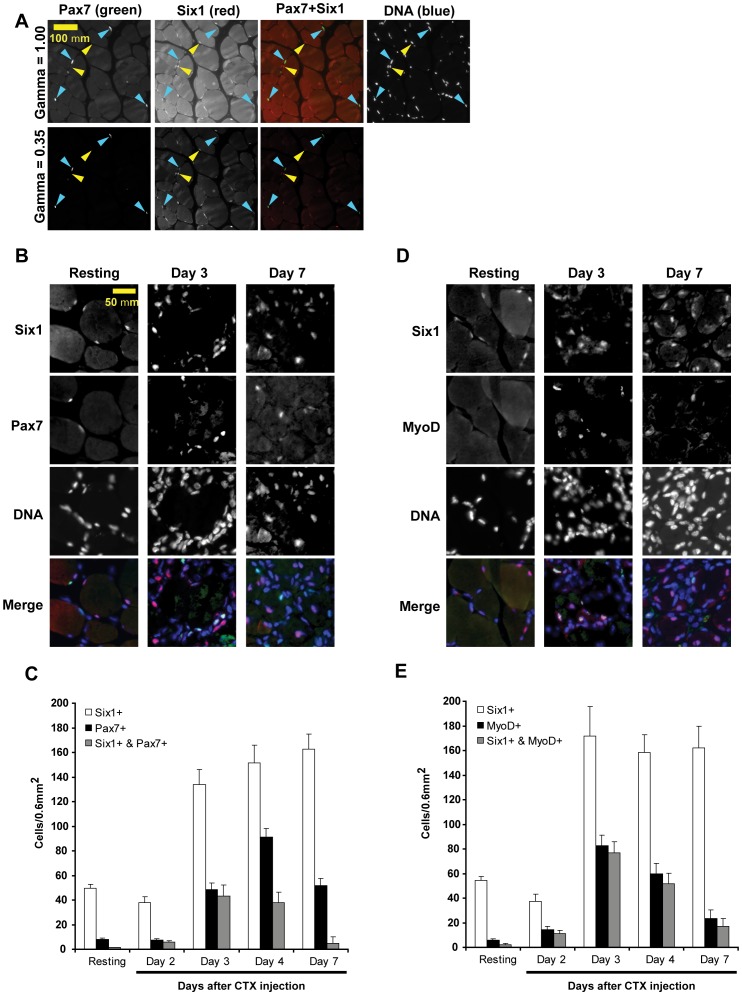
Six1 is expressed along with Pax7 and MyoD in activated satellite cells of regenerating muscles. **A)** Immunostaining of sections from paraffin-embedded resting TA muscles, using antibodies against Pax7 (green) and Six1 (red). Pax7-positive satellite cells are marked by blue arrowheads, while Six1-positive cells are marked by yellow arrows. Gamma settings were adjusted to 1.00 or 0.35 to increase the signal-to-noise ratio. DAPI was used as a counterstain to label nuclei. **B)** Immunostaining of frozen sections of resting TA, or muscles after 3 or 7 days following cardiotoxin injection, with antibodies against Six1 (red signal) and Pax7 (green signal). DAPI was used as a counterstain to label nuclei. **C)** Quantification of the anti-Six1 and anti-Pax7 staining signal in resting muscles and at 2, 3, 4 or 7 days post-injury, as shown in panel A. Bars indicate the average number of positively stained nuclei counted in 0.6 mm^2^ fields of view, using 5 mice per condition. Error bars, S.E.M. **D)** Immunostaining performed in samples identical as in A, but using antibodies against Six1 and MyoD. Magnification as shown in A. **E)** Quantification of the anti-Six1 and anti-MyoD staining as shown in C.

To study the function of Six1 during adult muscle regeneration, and to address the question whether it is involved in the regulation of MyoD expression, we started by determining the profile of Six1 and MyoD protein expression in the TA muscle of adult mice at various time points following injury. Intra-muscular injection of cardiotoxin was used as the injury model and immuno-fluorescence was performed on frozen muscle cross-sections to detect protein expression. First, sections were co-stained with antibodies against Six1 and the satellite cell marker Pax7 [7] (Fig. 1A and 1B). Six1 was undetectable in quiescent satellite cells from resting, uninjured muscles. Instead, we found the protein only in the myonuclei of myofibers. However, Six1 protein was clearly detected in Pax7-positive cells starting approximately three days following injury. This coincides with the surge in satellite cell numbers that occurs as these cells initiate proliferation following injury: at day 3 post-CTX, essentially all Pax7-positive cells are also Six1-positive. At later time-points, the situation is different: Six1 is highly expressed in the centrally-located nuclei of nascent or newly regenerated myofibers which are Pax7-negative, and Pax7-positive cells remain in high numbers but return to a state where they are Six1-negative.

MyoD has been reported to be expressed in satellite cells following injury [10], [11]. To determine if Six1 and MyoD are co-expressed, which is a logical requirement for a role of Six1 as regulator of MyoD expression, we co-stained regenerating muscle sections with antibodies against those two factors (Fig. 1C and 1D). We found that MyoD is undetectable in myonuclei or mononucleated cells from resting muscle, but that it is expressed along with Six1 at three and four days following CTX injection: virtually all MyoD-positive mononucleated cells are also Six1-positive at these two time-points. Seven days following injury, the numbers of MyoD-positive cells decrease but the cells remain mostly Six1-positive. Considering the staining pattern and the results in Fig. 1A, showing that at this time point the majority of Pax7-positive cells are Six1-negative, we reason that the double MyoD-positive/Six1-positive cells at day 7 are not satellite cells but rather represent the differentiating progeny of satellite cells (*i.e.* myocytes) or small, nascent myofibers. From these experiments, we conclude that Six1 is expressed in satellite cells as they become activated following injury, and that MyoD-positive satellite cells of regenerating muscle are also Six1-positive. This strong correlation between Six1 and MyoD expression is consistent with a role of Six1 as regulator of MyoD expression in activated satellite cells.

### Six1 is Required for MyoD Expression in Myoblasts

**Figure 2:**
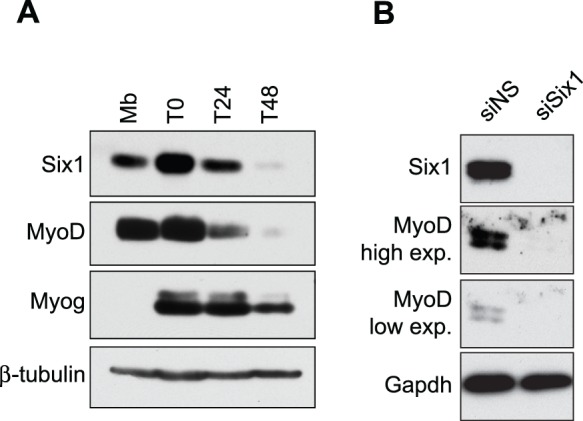
Six1 is expressed in primary myoblasts and is necessary for MyoD expression. **A)** Western blot on total protein lysates of primary myoblasts in growth phase (Mb), at confluence (T0), differentiated for 24 (T24) or 48 hours (T48). The antibodies used were anti-Six1, anti-MyoD and anti-myogenin. Anti-β-tubulin was used as a loading control. **B)** Western blot showing the expression of Six1, MyoD and GAPDH on total protein lysates of primary myoblasts in growth phase, 48 hours after their transfection with siRNA duplexes targeting Six1 (siSix1), or with a non-silencing siRNA (siNS). A low and a high film exposure are shown for the anti-MyoD western blot. Comparable results were obtained in three independent experiments.

To more directly address the question of whether Six1 is required for MyoD expression, we used primary myoblasts freshly isolated from adult mice as a model. Western blots reveal that proliferating primary myoblasts express appreciable levels of Six1 and MyoD, and that the levels decrease over time as the cells are induced to differentiate (Fig. 2A). We next tested whether MyoD protein levels in primary myoblasts depend on Six1 expression, by knocking down Six1 in growth phase myoblasts with siRNA against Six1 (siSix1), or non-silencing duplexes (siNS), and extracting total proteins from the cells 48 hours later, still in growth conditions. Western blot results in primary cells agree with those obtained with C2C12 cells (Fig. 2B): MyoD requires Six1 for its expression. An appreciable decrease in MyoD protein levels was also observed when Six1 expression was knocked-down using two different lentiviruses expressing distinct short hairpins against the Six1 mRNA (data not shown). These results indicate that MyoD expression in primary myoblasts depends on Six1 function, and suggest that Six1 performs a similar regulatory role *in vivo* in regenerating muscle.

### Six1 Binds the MyoD CER in Primary Myoblasts

**Figure 3:**
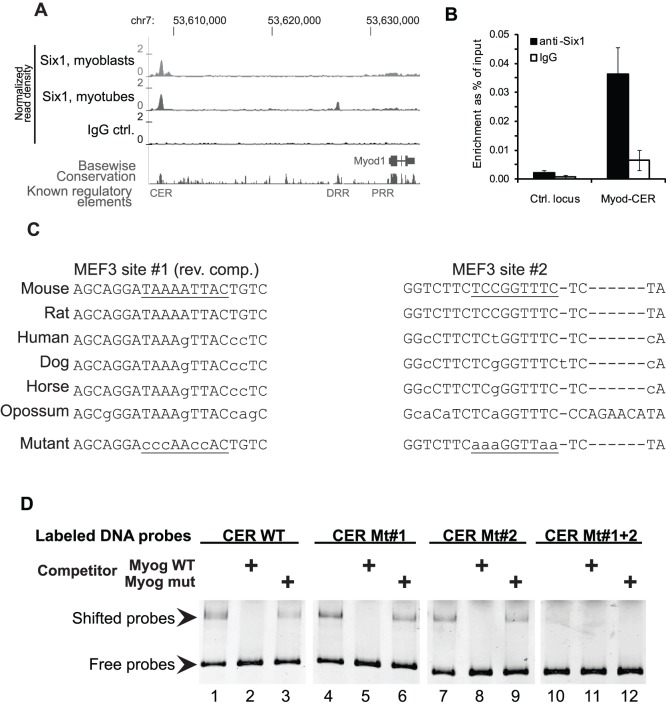
Six1 directly binds to the core enhancer region of MyoD at two conserved MEF3 sites. **A)** Profile of genomic binding of Six1 in primary myoblasts in growth phase, showing binding at the CER. The read density is expressed as reads per million mappable reads in bins of 25 base pairs above the read density in the input sample. The signal obtained with a non-specific antibody (non-immune rabbit IgG) is shown for comparison. **B)** Conventional gene-specific ChIP assays followed by real-time PCR were used to confirm the binding of Six1 to the CER, in proliferating myoblasts. n = 3 biological replicates (independent chromatin preparations). By one-tailed paired t test, the signal for anti-Six1 at the CER is significantly above that obtained on the negative control locus, and above that obtained at the CER with normal rabbit IgG, with p<0.05. Error bars, S.E.M. **C)** Sequences of the two MEF3 sites identified within the mouse core enhancer. The murine MyoD gene is on the+strand; the reverse-complement of site #1 is shown. Conservation across mammalian species is shown, along with the mutations created in the EMSA probes and reporter constructs. Positions in small script indicate divergent sequences using mouse as reference. Dots indicate sequence not shown; dashes indicate missing sequences in certain species. **D)** Direct binding of Six1 to the CER, shown by EMSA experiments using recombinant Six1 protein incubated with a wild-type CER probe, or with versions mutated at either or both MEF3 sites identified. Specificity of binding was assessed by competition with a 50-fold molar excess of unlabelled myogenin MEF3 site oligonucleotides, either wild-type sequence or mutated.

We have previously reported ChIP-on-chip analysis of Six1 binding in C2C12 myoblasts. We repeated similar experiments, this time using ChIP-sequencing (ChIP-seq) on chromatin prepared from primary myoblasts in their growth phase. A full analysis of these results will be described elsewhere (Y. Liu et al., in preparation). We analyzed the binding profile of Six1, reported as normalized read density, across the MyoD locus, and found that Six1 binds to the CER in myoblasts and in myotubes (Fig. 3A). We confirmed the binding of Six1 to this enhancer, using ChIP on independent primary myoblast chromatin preparations, thereby ruling out biases potentially introduced by the high-throughput sequencing approach (Fig. 3B). Examining the CER sequence, we found two MEF3-like elements that comply with the sequence elements we have shown Six1 is able to bind [45] (Fig. 3C). To determine if Six1 is able to directly bind to these elements, we performed electrophoretic mobility shift assays (EMSAs) using recombinant mouse Six1 produced in rabbit reticulocyte lysates and a fluorescently-labeled probe representing the full-length CER sequence. Competition using an excess of unlabelled oligonucleotides representing the MEF3 site of the Myogenin promoter [46], or a mutant version, was used to assess the specificity of protein binding. Six1 is indeed able to bind the CER directly and specifically, and this depends on the presence of at least one of the two MEF3 sites, since mutation of both sites was necessary to completely abolish protein binding in this assay (Fig. 3D).

### MyoD CER Activity Requires Six1 Binding

**Figure 4:**
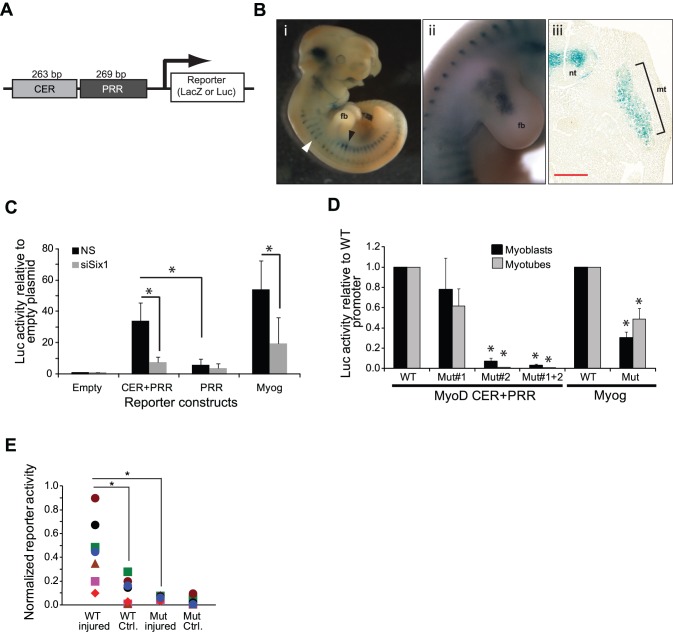
CER reporter constructs depend on Six1 expression and on Six1 binding sites for maximal activity. **A)** Schematic representation of the reporter constructs used for these experiments. The backbone for luciferase is pGL3-Basic, while that for LacZ is p1230. **B)** The murine CER+PRR LacZ construct drives reporter gene expression at the expected locations, in E11.5 transgenic founder mouse embryos. ii) The white arrowhead points to the dorsal part of the somites, with enhanced reporter activity. The black arrowhead points to the ventral part of the myotomes. fb, forelimb bud. ii) Forelimb bud signal on a different embryo. Signal is often seen in the hindlimb bud as well, on other embryos (not shown). iii) A cross-section at the inter-limb level reveals that the ventral signal seen in i) comes from the myotome (mt). Non-specific transgene expression is also detected in the neural tube (nt). **C)** CER enhancer activity depends on Six1 expression, as shown by promoter reporter assays performed in C2C12 myoblasts transfected first with the indicated luciferase reporter plasmids, and 24 hours later with the indicated siRNAs: control non-silencing or targeting Six1. The normalized luciferase activity readings are reported as fold over the numbers obtained with the empty pGL3-Basic plasmid and non-silencing siRNA. Bars represent the average of 3 biological replicates; error bars, S.E.M. Asterisks indicate significance (p<0.05) by two-tailed paired t test. **D)** CER enhancer activity depends on Six1 binding sites, as shown by reporter assays performed in primary myoblasts and myotubes. Myoblasts were transfected with the indicated reporter constructs, and either harvested as myoblasts or induced to differentiate for 48 hours prior to harvest. For comparison, the effect of MEF3 site mutation is also shown for the myogenin promoter. In each case, reporter activity is reported as fraction of the activity of the wild-type reporter. n = 3, asterisks indicate significance by two-tailed paired t test (p<0.05). Error bars, S.E.M. **E)** CER enhancer activity increases in regenerating muscle and this depends on the Six1 binding sites. Wild-type or MEF3-mutated reporter constructs were injected and electroporated in uninjured TA muscles, or in TA muscles 3 days post-injury by cardiotoxin injection. The transfected tissues were harvested 4 days later for luciferase assays. Values reported are normalized luciferase readings for each individual mouse leg harvested (n = 8, each depicted by a different symbol). Significance of reporter activity differences was assessed by Wilcoxon rank-sum test, with p<0.05 as threshold.

Considering that Six1 binds to the CER directly, we next aimed to determine if the enhancer’s activity depends on Six1. We cloned the murine CER and PRR elements one after the other in front of the LacZ and luciferase reporter genes (Fig. 4A). Since the murine CER has never been studied in this context, we first verified that our construct drives reporter gene expression faithfully in transgenic mouse embryos, based on the published literature on the human enhancer [14], [15], [47] and endogenous mouse gene [14]. Using LacZ as the reporter, we detected β-galactosidase activity in the somites and limb buds of mice at embryonic day 11.5 with a pattern similar to that described previously by others (Fig. 4A,B). Among other features, β-galactosidase staining anterior to the forelimb bud is most visible at the dorsal part of the myotome, while it is most obvious in the ventral myotome posterior to the forelimb bud. We next created a luciferase reporter construct, moving the mouse CER and PRR to the pGL3-Basic plasmid backbone. Transfection of this construct in C2C12 cells subsequently transfected with control or Six1-targeting siRNA duplexes revealed that the CER is active in C2C12 cells, and that this activity depends on Six1 expression (Fig. 4C). In contrast, a related construct containing only the PRR had a lower activity that was not dependent on Six1. We used the myogenin promoter, a well-known Six1 target gene, as a positive control in these assays; the Myog reporter behaved the expected way by showing a reduced activity upon Six1 knock-down. In order to determine which of the two MEF3 elements contributes to the enhancer activity, we also constructed mutant versions of the CER+PRR reporter where the two MEF3 sites were mutated singly or in combination. The reporters were transfected in primary myoblasts, and the cells were harvested in growth phase or after two days in differentiation medium to induce myotube formation. The results show that MEF3 site #2 is by far the most active since its mutation caused the greatest reduction in luciferase activity (Fig. 4D). In contrast, site #1 mutation had a slight but not statistically significant effect on reporter activity, both in the contexts of the wild-type or mutated site #2. Finally, to assess the *in vivo* significance of the Six1 binding sites on CER activity, we transfected resting or regenerating TA muscles with the wild-type or double MEF3 sites mutant CER+PRR luciferase constructs, and determined reporter activity four days later. We found that the CER reporter activity is significantly higher in regenerating muscle, and that this depends on the presence of the Six1 binding sites (Fig. 4E). Although the luciferase activity may originate from various cell types in addition to satellite cells, our results indicate that the CER of MyoD is a functional binding site for Six1 and that enhancer activity depends on Six1 function.

### Six1 is Necessary for Proper Chromatin Structure and for MyoD Binding at the CER

**Figure 5:**
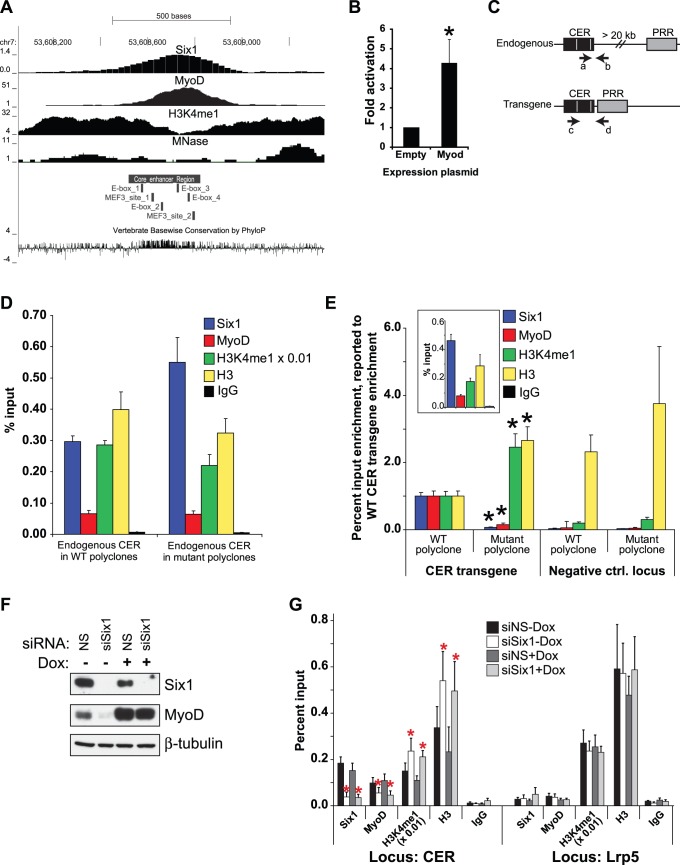
Chromatin structure and MyoD binding at the CER depend on Six1 function. **A)** Genomic binding profiles of Six1 in primary myoblasts (this study), and of MyoD [^22^], H3K4me1 and mononucleosomes [^49^] in C2C12 myoblasts. The position of the MEF3 sites and of three E-boxes (CANNTG) is also shown. **B)** Over-expression of MyoD in HEK293T cells leads to activation of the CER+PRR luciferase reporter gene. The results are reported over the luciferase activity obtained with an empty expression plasmid, and reflect the average of three independent replicates. Asterisk, p<0.05 by one-tailed paired t test. **C)** Schematic representation of the PCR strategy used to distinguish the endogenous CER and the exogenous CER+PRR transgene. Primers a and b together can only amplify the endogenous CER sequences, while primers c and d will only give an amplification product on the CER+PRR reporter gene, since the endogenous CER and PRR are separated by more than 20 kb of sequence. The white bars in the CER represent the two Six1 binding sites. **D) and E)** ChIP assays performed on chromatin from stable polyclones of the CER+PRR reporters (wild-type or double MEF3 sites mutant). **D)** The real-time PCR quantities of the endogenous CER (primers a+b) were expressed as percentage of input chromatin. The level of binding of Six1, MyoD, H3K4me1 and H3 on the endogenous CER is not significantly different in wild type and mutant polyclones. Because the H3K4me1 signal is very high, we divided the values plotted for this mark by 100. **E)** The real-time PCR quantities of the transgene (primers a+c) were expressed as percentage of input chromatin and normalized over the quantities of the endogenous CER locus (primers a+b, used as internal control). Enrichment at a control locus (not targeted by Six1 or MyoD) is also given, in each set of polyclones. The results are reported as a fraction of the enrichment obtained on the wild-type CER+PRR construct. The inset shows the enrichment of Six1, MyoD, H3K4me1 and H3 as percentage of input on the wild type transgene. n = 3 replicates for each reporter gene construct; bars: S.E.M. Asterisks, p<0.05 by unpaired two-tailed t test. **F)** Western blot showing the levels of Six1 and MyoD in C2iFRT-FL-MyoD cells transfected with the control (siNS) or Six1 knock-down (siSix1) siRNA duplexes, and treated or not with doxycycline to induce MyoD expression. Beta-tubulin is shown as loading control. **G)** ChIP assays performed on chromatin isolated from C2iFRT-FL-MyoD cells treated as in F. The enrichment is shown as percent of input chromatin. Because the H3K4me1 signal is very high, we divided the values plotted for this mark by 100. The Lrp5 locus serves as a control locus not targeted by Six1 or MyoD. Asterisks indicate p<0.05 by one-tailed paired T test.

Since Six1 has been shown to interact with proteins that can alter chromatin structure [37], [48], we reasoned that chromatin remodeling might underlie the regulatory role of Six1 at the CER. We analyzed ChIP-seq data for the H3K4me1 (mono-methylated histone H3 lysine 4) in C2C12 myoblasts [49], since this is a mark associated with transcriptional enhancers [50]. Figure 5A shows the Six1, MyoD, H3K4me1 and mono-nucleosomes location profiles in myoblasts. Interestingly, the peak of Six1 binding is located very close to the peak of MyoD binding [22]. Furthermore, Six1 localizes to an area possessing the typical enhancer element architecture where a nucleosome poor domain is flanked on both sides by nucleosomes bearing the H3K4me1 mark. To determine if Six1 is responsible for establishing this enhancer structure, we first devised a strategy that involves ChIP assays on genome-integrated CER+PRR reporter genes. We stably transfected C2C12 myoblasts with our CER+PRR luciferase constructs, in their wild-type or double MEF3-site mutant versions, by co-transfecting either plasmid with limited amounts of a puromycin-resistance gene expression plasmid. Drug-resistant cells were pooled together to constitute either wild-type or double MEF3-site mutant CER “polyclones”. Using quantitative PCR, we ensured that the wild-type and mutant polyclones contained equal numbers of transgenes (data not shown). Comparing ChIP assay results between the wild-type and mutant polyclones allowed us to ascertain the role of Six1 in establishing the CER enhancer architecture without having to use Six1 loss-of-function. This is important because we have shown that Six1 controls MyoD expression (Fig. 2) and because MyoD controls its own transcription [51]. In fact, MyoD can activate a transiently-transfected CER-luciferase reporter transgene in heterologous cells (Fig. 5B). We designed PCR primer pairs to be used after ChIP that would allow us to distinguish protein binding at the endogenous CER and at the CER transgene separately (Fig. 5C). We first performed ChIP assays using antibodies against Six1, MyoD, H3K4me1 or an antibody recognizing all forms of histone H3, and performed quantitative PCR for the endogenous CER locus (primers a and b). This allowed us to confirm that the presence of these proteins or histone marks at the endogenous locus is not overtly different in the wild-type and the mutant CER polyclones (Fig. 5D). On the same ChIP samples, we then performed quantitative PCR using primers c and d, to detect specifically enrichment at the CER transgene. All proteins or marks are detected at the wild-type CER transgene as at the endogenous enhancer (Fig. 5E, inset). However, binding of Six1 to the double MEF3-site mutant transgene is greatly impaired, as expected (Fig. 5E). Moreover, MyoD recruitment to the MEF3-site mutant is also severely diminished. Strikingly, the presence of H3K4me1, and of nucleosomes in general (evidenced with the histone H3 antibody), is markedly increased when Six1 binding is prohibited.

Secondly, in order to confirm that loss of Six1 causes a remodeling of chromatin at the endogenous CER, we performed the knock-down of Six1 in a C2C12 cell line where the expression of MyoD can be induced by treatment with doxycycline (C2iFRT-FL-MyoD cells). Using this system, induction of exogenous MyoD expression allows us to maintain MyoD protein levels in the absence of Six1. As such, we can determine the impact of loss of Six1 without the complication of concomitant loss of MyoD protein. Western blot analysis confirmed that Six1 knock-down leads to lower MyoD expression levels, and revealed that treatment with doxycycline indeed rescues the MyoD expression defect (Fig. 5F). ChIP assays were then performed on similar samples using antibodies against Six1, MyoD, H3K4me1 and H3 (and normal IgG). As expected, Six1 binding decreases to background levels after its knock-down. Furthermore, we detected lower recruitment of MyoD at the CER after Six1 knock-down, which is consistent with the lower MyoD protein levels in this condition. Interestingly, as was the situation with the transgenic assays (Fig. 5E), the results showed that the global abundance of nucleosomes, and of nucleosomes bearing the H3K4me1 mark, increases when Six1 is knocked down (Fig. 5G). Importantly, the impact of Six1 knock-down was not due to the lower expression of MyoD since rescue by over-expression of Flag-MyoD failed to return the H3 or H3K4me1 signals to normal. Finally, our results confirmed that MyoD binding to the CER requires the presence of Six1, since induction of exogenous MyoD expression by the addition of doxycycline does not rescue its ability to bind to this enhancer. None of these effects were detected at the Lrp5 locus, where a robust H3K4me1 signal can be detected but where neither Six1 nor MyoD bind. These results combined indicate that Six1 binding is required for establishing or maintaining the appropriate structure of chromatin at the CER and for allowing MyoD to bind its enhancer.

## Discussion

We have shown that Six1 is expressed in satellite cells of adult muscle in regeneration, and that its expression and function are consistent with its role in regulating MyoD expression: the Six1 protein is detected in activated satellite cells, and its expression coincides with the presence of MyoD. Further, the expression of MyoD is attenuated in myoblasts where Six1 expression is knocked-down, suggesting that Six1 accomplishes a similar function in activated satellite cells. We have also demonstrated that Six1 exerts its function *in vitro* and *in vivo* through two MEF3 sites within the CER enhancer of MyoD, and that it acts at least in part by contributing to the specific chromatin architecture of the enhancer. We reason that the action of Six1 towards chromatin remodeling contributes to the action of additional transcription factors, such as MyoD itself, which we have shown can directly activate transcription from its CER.

Our findings suggest that Six1 exerts its effect on MyoD expression via the core enhancer region: the CER activity depends on Six1 and on its binding sites, in cultured cells and in regenerating muscle. We cannot rule out the involvement of other regulatory regions in the regulation of MyoD expression. However, our results show that the increased CER *in vivo* activity in regenerating muscle parallels the increased expression levels of MyoD after injury, so the CER appears a relevant enhancer to control MyoD upregulation in regenerating muscle. We and others have found that Six1 can also bind to the DRR enhancer [23], but we found that this binding is limited to primary myoblasts that are undergoing differentiation: binding of Six1 to the DRR in proliferating cells was undetectable. The situation is similar for MyoD, since it binds its DRR only in myotubes, not in myoblasts [22]. These observations regarding transcription factor binding at the DRR are in line with findings made with transgenic reporter mouse embryos, which have revealed that DRR-LacZ reporter genes are mostly active in differentiated muscle cells [16], [17]. Based on this, we conclude that of those two enhancers, the CER is the most relevant to the induction of MyoD expression in activated satellite cells.

A mechanistic clue as to how Six1 regulates MyoD expression came from examining the structure of chromatin at the CER. This enhancer, like many others, is characterized by a relative paucity of nucleosomes and by its flanking by nucleosomes bearing the H3K4me1 mark. In the absence of MEF3 sites, Six1 binding fails to occur and the structure of chromatin at the enhancer is altered: nucleosomes are more abundant, and they bear the H3K4me1 mark. This situation is reminiscent to that recently reported for Pax7 and Tpit target genes in the pituitary gland: the pioneering action of Pax7 is associated with a conversion of its target enhancers from a unimodal H3K4me1 distribution centered at its binding sites, to one that is bimodal, flanking the Pax7 binding sites on both sides and which allows Tpit binding [52]. We observed a similar situation in muscle precursor cells: our results reveal that in the absence of Six1, the MyoD protein is unable to bind its own enhancer. Based on our observations, we therefore propose that Six1 might analogously act as a pioneer factor that enables MyoD recruitment to the CER, by establishing a chromatin environment that enables MyoD to access DNA. The concept of pioneer factor-facilitated MyoD binding at target genes has a precedent: the homeodomain factor Pbx1 has been shown to constitutively bind the Myogenin promoter, and to facilitate the recruitment of MyoD at that locus upon differentiation [53], [54]. Considering that Six1 is known to directly interact with components of the nucleosome-displacing SWI/SNF complex during inner ear neurogenesis, it is possible that Six1 might contribute to recruit an analogous complex at the CER in activated satellite cells. This would serve to open up the chromatin structure and allow MyoD to bind its own enhancer. According to this model, the effect of Six1 on MyoD induction can occur only in the presence of a certain amount of pre-existing MyoD protein. Assuming that MyoD protein levels are absolutely null in quiescent satellite cells, the initial appearance of MyoD protein would be Six1-independent, and instead could rely on other mechanisms such as microRNA regulation as has been shown for the related gene Myf5 in satellite cells [9]. Once MyoD protein levels reach a certain threshold, a Six1- and MyoD-dependent boost of MyoD gene transcription would occur. Our observation that MyoD can indeed activate transcription from its CER enhancer, together with the well-established fact that MyoD regulates its own expression, is consistent with such a model. Another, non-mutually exclusive possibility is that Six1 function at the CER permits the binding of other transcription factors, in addition to MyoD.

Our model that Six1 functions at the CER by facilitating the recruitment of MyoD through the remodeling chromatin at this locus adds to the possible mechanisms by which chromatin regulation controls muscle cell differentiation [55]. We have previously reported that close to 40 percent of the loci bound by Six1 overlap to a highly significant degree with MyoD binding sites in C2C12 myoblasts [38], which suggests that other MyoD targets may also be regulated in a similar fashion by Six1. It will therefore be interesting to determine whether this mechanism for the combinatorial regulation of transcription of muscle genes is a general feature of MyoD-Six1 joint targets.

Yajima et al. have previously reported that Six1 is expressed in satellite cells: they found that Six1 was present in quiescent satellite cells of resting muscle, and in those of muscles in regeneration [56]. In addition, Le Grand et al. have detected the presence of the Six1 protein in seemingly quiescent satellite cells on explanted myofibers [23]. We failed to detect Six1 protein expression in quiescent satellite cells on frozen muscle sections, and we postulate that this could be due to technical differences (e.g. sensitivity of the method and/or specificity of the immunological reagents used). Based on previous reports, we also surmise that the isolation of single myofibers may constitute a stress sufficiently strong to cause the activation of satellite cells [9], [57]. Nevertheless, our main conclusions that Six1 protein expression increases following activation, and that the number of Six1-expressing cells increases as well following injury, are consistent with the previous reports. We found that the majority of MyoD-positive cells maintain Six1 expression during satellite cell activation (2, 3, 4 days after injury) before MyoD expression goes down with myofiber maturation (7 days after injury), suggesting that Six1 contributes to muscle regeneration by augmenting and maintaining MyoD expression in the satellite cells in their proliferative and early differentiation phases. This is consistent with the results of Le Grand, who showed that explanted myofibers of satellite cell-specific Six1 knock-outs express less MyoD-positive satellite cells [23]. Our molecular analyses suggest that Six1 favors this commitment of muscle stem cells by facilitating MyoD self-regulation, at least in part by enabling MyoD binding and participating in the remodeling of chromatin at the core enhancer region.

## Supporting Information

Table S1 Binding profile of Six1 in primary myoblasts at the MyoD locus. The data are in WIG format and can be uploaded as custom tracks on the UCSC genome browser, using the mouse mm9 genome release. The file contains the read density obtained with anti-Six1 and the read density obtained using normal rabbit IgG as negative control.
(TXT)
